# Impact of thyroid hormone replacement therapy on the course and functional outcome of aneurysmal subarachnoid hemorrhage

**DOI:** 10.1007/s00701-024-06118-7

**Published:** 2024-06-03

**Authors:** Maryam Said, Meltem Gümüs, Christoph Rieß, Thiemo Florin Dinger, Laurèl Rauschenbach, Jan Rodemerk, Mehdi Chihi, Marvin Darkwah Oppong, Philipp Dammann, Karsten Henning Wrede, Ulrich Sure, Ramazan Jabbarli

**Affiliations:** 1 Department of Neurosurgery and Spine, Surgery University Hospital of Essen, 45147 Essen, Germany; 2 Department of Neurosurgery and Spine Surgery, Evangelisches Krankenhaus Oldenburg, Essen, Germany; 3 Center for Translational Neuro- & Behavioral Sciences (C-TNBS), University Duisburg, Essen, Germany

**Keywords:** Hypothyroidism, Outcome, Prediction, Subarachnoid hemorrhage

## Abstract

**Background:**

Thyroid hormones were reported to exert neuroprotective effects after ischemic stroke by reducing the burden of brain injury and promoting post-ischemic brain remodeling.

**Objective:**

We aimed to analyze the value of thyroid hormone replacement therapy (THRT) due to pre-existing hypothyroidism on the clinical course and outcome of aneurysmal subarachnoid hemorrhage (SAH).

**Methods:**

SAH individuals treated between January 2003 and June 2016 were included. Data on baseline characteristics of patients and SAH, adverse events and functional outcome of SAH were recorded. Study endpoints were cerebral infarction, in-hospital mortality and unfavorable outcome at 6 months. Associations were adjusted for outcome-relevant confounders.

**Results:**

109 (11%) of 995 individuals had THRT before SAH. Risk of intracranial pressure- or vasospasm-related cerebrovascular events was inversely associated with presence of THRT (*p* = 0.047). In multivariate analysis, THRT was independently associated with lower risk of cerebral infarction (adjusted odds ratio [aOR] = 0.64, 95% confidence interval [CI] = 0.41–0.99, *p* = 0.045) and unfavorable outcome (aOR = 0.50, 95% CI = 0.28–0.89, *p* = 0.018), but not with in-hospital mortality (aOR = 0.69, 95% CI = 0.38–1.26, *p* = 0.227).

**Conclusion:**

SAH patients with THRT show lower burden of ischemia-relevant cerebrovascular events and more favorable outcome. Further experimental and clinical studies are required to confirm our results and elaborate the mechanistic background of the effect of THRT on course and outcome of SAH.

**Supplementary Information:**

The online version contains supplementary material available at 10.1007/s00701-024-06118-7.

## Introduction

Although aneurysmal subarachnoid hemorrhage (SAH) is less frequent than ischemic stroke, its considerable morbidity and mortality and higher prevalence in young and middle-aged adults carry a substantial public health impact [31, 32, 36]. Even with modern day neuro-intensive monitoring and guideline-based treatment concepts, early and delayed SAH complications like pathologically increased intracranial pressure (ICP) and cerebral infarcts along with other adverse events still contribute to a poor outcome in patients with ruptured aneurysms [30].

In an effort to prevent or at least reduce the burden of outcome-relevant complications, numerous endeavors have been made to optimize SAH management in intensive care units over recent decades. Specifically, both experimental and clinical research have explored the potential of various pharmacological agents for the medical treatment of SAH [7]. While nimodipine remains the only universally recommended drug for SAH patients according to guidelines [16, 39], there are reports suggesting potential advantages from the use of other pharmacological substances, such as clazosetran [34], aspirin [10], statins [45] or magnesium [3]. In this context, evaluation of the potential impact of regular medication taken by SAH patients for pre-existing comorbidities, as well as the influence of these comorbidities on the course and outcome of SAH, is of particular interest [19]. Unfortunately, data on the therapeutic effects of pharmacological substances on SAH are scarce and require further evaluation.

Thyroid hormones are essential representatives of the endocrine system, exerting a strong impact on cellular growth, differentiation, and energetic regulation in various organ systems [43]. Normal levels of thyroid hormones are required for normal anabolism—both excess and deficiency exert an inhibitory effect [4]. Thyroid hormones have been shown to play a particular role in many acute conditions. So, preclinical and clinical studies reported that thyroid hormones administration were associated with reduced infarct volume and lesser neurological deficit, suggesting that thyroid hormones have a potential role in reducing ischemic brain damage [5]. Although thyroid hormones alterations have also been described after SAH [5, 6, 25], the relevance of thyroid metabolism and thyroid hormone replacement therapy (THRT) for outcome of SAH patients remains unclear.

Therefore, the current large retrospective study is the first to analyze the value of THRT due to pre-existing hypothyroidism on the clinical course and outcome of SAH.

## Methods

This is a retrospective single-center study based on the institutional aneurysm database. All consecutive cases with aneurysmal SAH treated in our clinic between January 2003 and June 2016 were included in our analyses. There were no restrictions on inclusion criteria regarding the patients’ baseline and initial SAH characteristics. The local institutional ethics committee approved our study (registration number: 15–6331-BO). Additionally, it is registered in the German clinical trial register (DRKS, Unique Identifier: DRKS00008749). All persons or their relatives gave their informed consent within the written treatment contract signed on admission to our institution. All the methods are in accordance with relevant institutional guidelines and regulations.

### Management of SAH

Patients were admitted to our neurosurgical intensive care unit and diagnostic digital subtraction angiography (DSA) of the cerebral vessels was performed. Patients were treated surgically or endovascularly after interdisciplinary consensus with the neuroradiologists, commonly < 24 h after admission. Baseline conservative management included nimodipine and normovolemia. In the absence of medical contraindications (such as in the case of blood-thinning and blood pressure-controlling medication), the chronic medication taken by patients prior to SAH was continued unchanged during SAH treatment. Acute hydrocephalus was treated with external ventricular drainage (EVD), which also allowed for continuous ICP-measurement. Daily transcranial Doppler (TCD) sonography was performed to monitor cerebral vasospasms. Endovascular spasmolysis was performed for persistent vasospasms in TCD in unconscious patients and/or development of delayed ischemic neurological deficit (DIND) not attributable to other causes than vasospasm, after confirmation of vasospasms in the DSA. Persistent ICP > 20 mmHg was treated by deep sedation and relaxation, osmotic therapy, and forced cerebrospinal fluid (CSF) diversion. If despite these measures ICP remained pathologically elevated, a decompressive craniectomy (DC) was performed. Chronic hydrocephalus was treated with a permanent CSF shunt in the course of disease. Computed tomography (CT) scans of the head were performed upon admission, before and after every cranial intervention and with every neurological deterioration. For the studied years, no significant modifications in this management protocol were made.

### Data management

Data on the baseline demographic parameters, initial radiographic and clinical severity of SAH, treatment modality, adverse events in the course of hospitalization and functional outcome after SAH were collected from the institutional aneurysm database fed from the patients’ electronic health records. For the assessment of radiographic parameters, all initial and follow-up imaging data stored in the institutional picture archiving and communication system were reviewed by the senior author (R.J.) blinded at that time for any clinical information, as previously reported [17, 18]. Data on patients’ medical history and regular medication were based on the institutional standardized anamnesis form filled out obligatorily at admission. In unconscious patients and in the events of uncertainties, the family doctors and the next of kin were surveyed for completing the records. The data were analyzed with regard to THRT in the context of pre-existing hypothyroidism. Clinical severity of SAH was expressed as the World Federation of Neurosurgical Societies (WFNS) grade [41]. Radiographic severity was measured by means of the original Fisher score [13] and the Subarachnoid Early Brain Edema Score (SEBES) [1]. For further analyses, the SAH severity scales were dichotomized at commonly used cut-offs: WFNS = 4–5 vs 1–3, Fisher = 3–4 vs 1–2, and SEBES = 3–4 vs 0–2. Functional outcome was assessed by the modified Rankin scale (mRS) [40], which was routinely performed 6 months after ictus in our outpatient department. Cases with mRS > 3 were considered patients with unfavorable outcome. All follow-up CT scans up to 6 weeks after SAH were reviewed by the senior author (R.J.) for occurrence of new hypodensities attributable to cerebral infarctions. Other parameters and events of interest included: aneurysm rebleeding, occurrence of acute hydrocephalus, ICP–related problems (requiring conservative treatment and/or need for DC), occurrence of vasospasms in TCD, clinical deterioration of delayed cerebral ischemia (DCI) attributable as DIND (according to the current definitions [42]), shunt dependency, occurrence of systemic infections and fever (a mean daily body temperature > 38.0°) during the initial hospital stay. Development of ICP- (conservative treatment and/or DC) and vasospasm- (DIND or in TCD measurements) related complications during SAH were also assessed as a cumulative variable defined as “cerebrovascular events” (no vasospasm / no ICP increase vs. one of these events [vasospasm or ICP > 20 mmHg] vs. both events [vasospasm and ICP > 20 mmHg]). The occurrence of fever was additionally analyzed depending on the presence of systemic infections (fever with and without systemic infections).

### Study endpoints and statistical analyses

We analyzed the effect of the presence of hypothyroidism in the medical history and THRT in relation to the following primary endpoints: 1) development of new cerebral infarcts in the follow-up CT scans, 2) in-hospital mortality and 3) unfavorable outcome at 6 months after ictus. Secondary endpoints included the above-mentioned adverse events after aneurysm treatment. To measure the possible effect of THRT on the occurrence of cerebrovascular complications relevant for DCI development [46], the occurrence of ICP and vasospasm in TCD were grouped as “DCI-relevant cerebrovascular events” and also analyzed as secondary study endpoint.

Statistical analyses included the correlations between THRT with the baseline parameters and the clinical events as outlined in the study endpoints. In the univariate analysis, associations between THRT and the continuous variables were calculated using the Student’s t-test for normally distributed continuous data and the Mann–Whitney U-test for non-normally distributed data. For categorical variables, the Chi-squared test or Fisher’s exact test were used, as appropriate. In the final multivariate analyses, associations between THRT and the study endpoints were adjusted for major endpoint-relevant confounders: patients age, sex (due to strong association with hypothyroidism), baseline SAH severity (WFNS / Fisher grades at admission), acute hydrocephalus, treatment modality and occurrence of aneurysm rebleeding. Statistical analysis was performed using SPSS statistical software (version 25, SPSS Inc., IBM). Missing values were replaced using multiple imputation (data on the portion of missing variables is presented in Supplementary Table S1). As per standard procedure, five imputations were computed using a model accounting for all variables included in the multivariate analysis. Correlations with a *p*-value of ≤ 0.05 were considered statistically significant.

## Results

In total, 995 SAH patients were treated during the observational period, and, therefore, included in the study. The major baseline characteristics of the cohort are presented in Table 1. The mean age of the final cohort was 54.7 years (± 14), 667 patients were females. Microsurgical clipping and endovascular treatment of the ruptured aneurysm was performed in 365 and 579 cases respectively, 51 SAH patients underwent no aneurysm treatment due to poor initial condition and/or patients’ living will (see supplementary table S2 for comparison of SAH patients with/without aneurysm treatment).

**Table 1:** Baseline characteristics and adverse events during SAH

Parameter	Number (%^*^) or Mean (± SD)
*Demographics*	
Age (years)	54.7 (± 14)
Female	667 (67.0%)
*Initial SAH characteristics*	
WFNS grade 4–5	413 (41.5%)
SEBES grade 3–4	427 (48.0%)
Fisher grade 3–4	753 (86.3%)
Clipping	365 (36.7%)
Rebleeding	58 (5.8%)
Acute hydrocephalus	694 (69.7%)
*Complications after SAH*	
ICP increase > 20 mmHg	440 (44.2%)
DC	257 (25.8%)
DIND	256 (30.7%)
TCD vasospasms	442 (51.8%)
Shunt dependency	279 (33.0%)
Fever	720 (78.9%)
Systemic infections	375 (41.9%)
*Outcome after SAH*	
Cerebral infarcts	477 (48.3%)
In-hospital mortality	180 (18.1%)
Unfavorable outcome^†^	349 (37.7%)

Abbreviations: *SAH* aneurysmal subarachnoid hemorrhage, *WFNS* World Federation of Neurosurgical Societies, *SEBES* Subarachnoid hemorrhage Early Brain Edema Score, *DC* Decompressive craniectomy, *ICP* intracranial pressure, *DIND* delayed ischemic neurological deficit, *TCD* transcranial Doppler sonography. * percentages were calculated according to the number of cases with known values; † modified Rankin scale > 3 6 months after ictus

### THRT due to pre-existing hypothyroidism: prevalence, baseline characteristics and the relation to the study endpoints

**Fig. 1:**
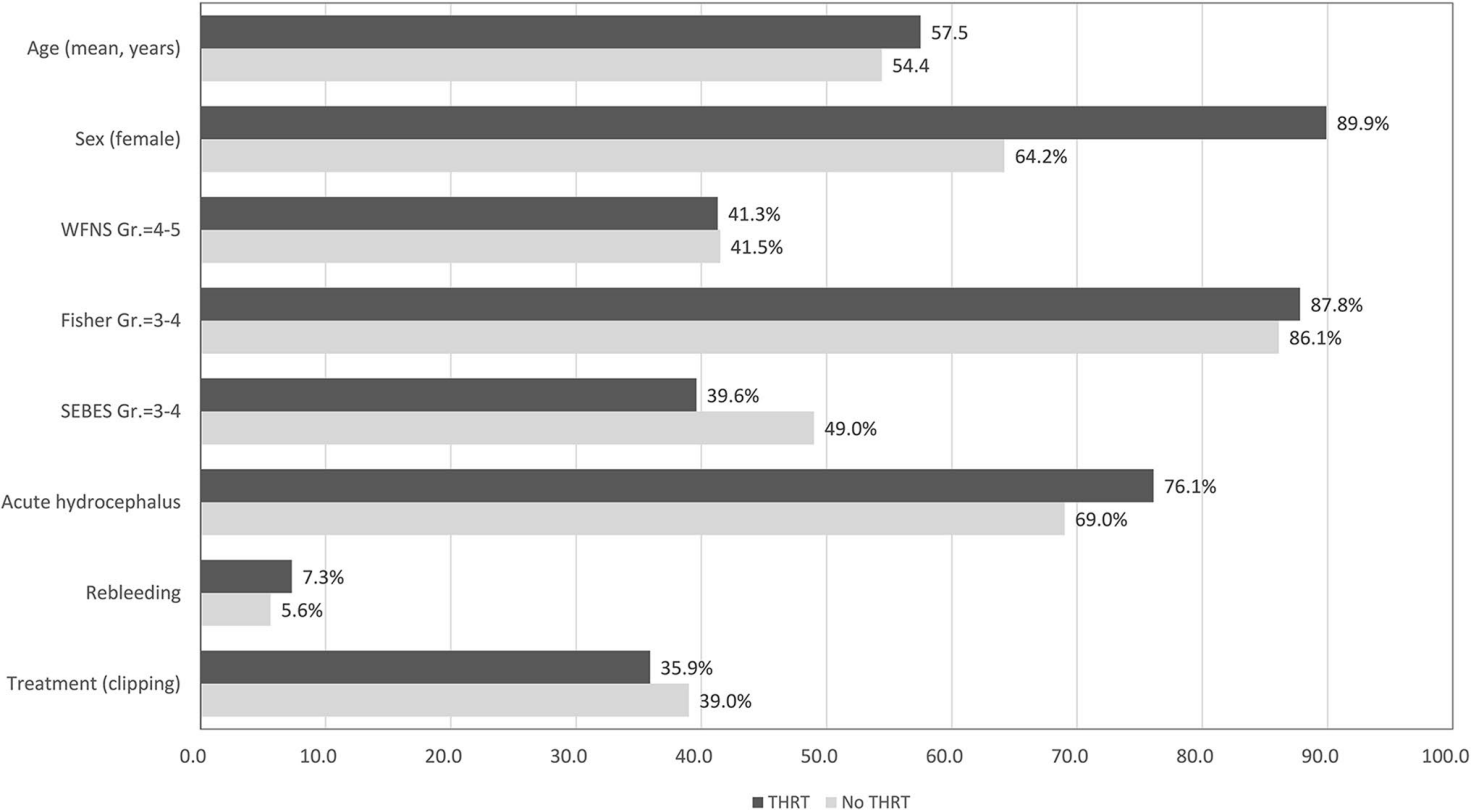
Comparison of baseline demographic and clinical parameters in patients with/without THRT due to pre-existing hypothyroidism. Females were significantly more often affected by hypothyroidism (*p* < 0.0001). The mean age in individuals with THRT is significantly higher (*p* = 0.013). With regard to remaining characteristics, the differences did not reach the statistical significance in the univariate analyses (see the Supplementary Table S2)

In the final cohort, 109 (11%) individuals had a pre-clinically diagnosis of hypothyroidism for which they were taking replacement therapy at the time of admission for SAH. The mean age in the cohort without known hypothyroid dysfunction was 54.4 years. In the THRT subgroup, the mean age was significantly higher at 57.5 years (*p* = 0.013). A remarkably higher percentage of patients with THRT was female (89.9% vs. 64.2%, *p* < 0.0001). In both groups, the number of patients affected by a severe SAH were similar regarding the WFNS and Fisher grades. Individuals with THRT showed a non-significantly lower rate of the initial brain edema on admission CT scans (SEBES = 3–4: 39.6% vs. 49%, *p* = 0.085, see Fig. 1 and Supplementary Table S2 with comparison of all demographic and clinical parameters).

Regarding the primary study endpoints, there were less cases with unfavorable outcome (28% vs 38.9%, *p* = 0.038), and a lower rate of cerebral infarcts (40.7% vs. 49.2%, in the univariate analysis: *p* = 0.103) in SAH patients with THRT due to hypothyroid dysfunction. The rate of in-hospital mortality was comparable (15.6% vs. 18.4%, *p* = 0.513).

**Fig. 2:**
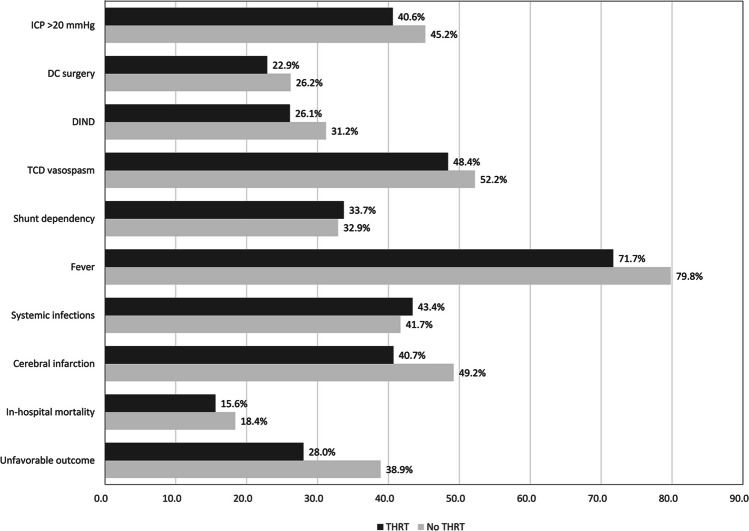
Comparison of adverse events and outcome in patients with/without THRT. Intracranial pressure (ICP)-related complications (both need for conservative treatment as for decompressive craniectomy (DC) surgery) are less prevalent in patients with THRT due to pre-existing hypothyroidism. Also, transcranial Doppler sonography (TCD) vasospasms and delayed ischemic neurological deficit (DIND) are less common in this subgroup, as was the occurrence of fever. Finally, the primary study endpoints (cerebral infarctions, in-hospital mortality and unfavorable outcome) were all less frequent in patients with THRT

When comparing the adverse events (see Fig. 2 and Supplementary Table S3), the univariate analysis did not show significant differences between the groups with regard to the risk of ICP increase requiring conservative treatment (40.6% vs. 45.2%, *p* = 0.409) and DC (22.9% vs. 26.2%, *p* = 0.562), occurrence of TCD vasospasm (48.4% vs. 52.2%, *p* = 0.507), DIND (26.1% vs. 31.2%, *p* = 0.340), and fever (71.7% vs. 79.8%, *p* = 0.068). The rates of shunt dependency (33.7% vs. 32.9%, *p* = 0.907) and systemic infections (43.4% vs. 41.7%, *p* = 0.747) were also comparable.

**Fig. 3:**
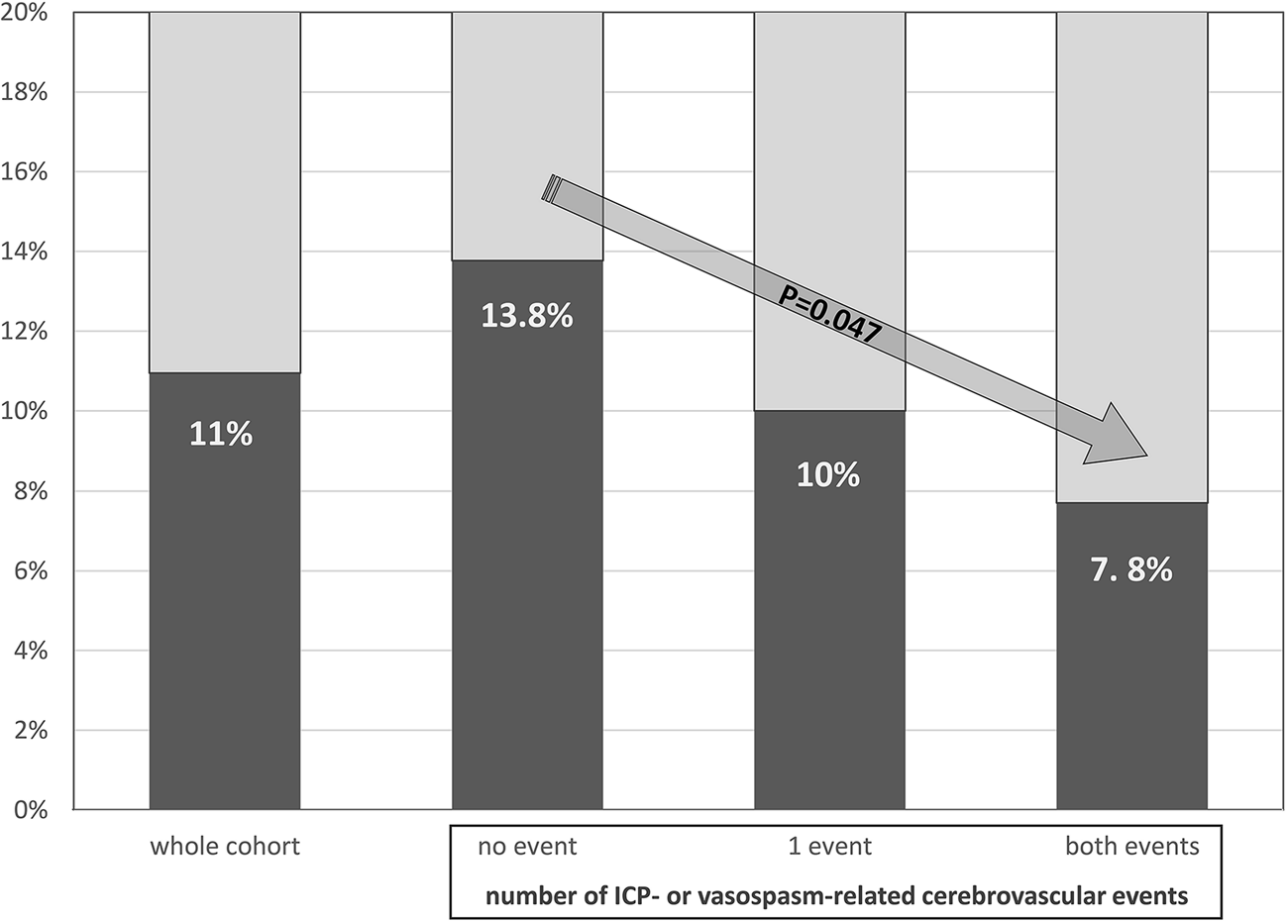
Rates of THRT due to pre-existing hypothyroidism compared for the number of intracranial pressure (ICP)- or vasospasm related cerebrovascular adverse events. There is a significant correlation between the THRT and these adverse events. The more the number of adverse events, the less the prevalence of THRT

When summarizing the ICP- and TCD-related complications into a group of “DCI-relevant cerebrovascular events”, SAH patients without these events showed the highest prevalence of THRT (13.8%), followed by the cases with only one cerebrovascular event (10%). The lowest rate of THRT (7.8%) was observed among individuals with ICP- and vasospasm-related adverse events (*p* = 0.047, Fig. 3). Of note, there was an increasing rate of cerebral infarctions with increasing number of DCI-relevant cerebrovascular events: 27%, 54.3% and 78.9% infarctions in SAH individuals without (no ICP increase / no vasospasm in TCD), with one (ICP increase or TCD vasospasm) and both (ICP increase and TCD vasospasm) adverse events respectively (*r* = 0.313, *p* < 0.0001).

### Multivariate analyses for the primary and secondary study endpoints

In the final analyses adjusted for outcome-relevant confounders (Table 2), THRT was independently associated with a lower risk of cerebral infarcts (adjusted odds ratio [aOR] 0.64, 95% confidence intervals [CI] 0.41–0.99, *p* = 0.045) and unfavorable outcome (aOR 0.50, 95% CI 0.28–0.89, *p* = 0.018) after SAH. There was no impact of THRT on the risk of in-hospital mortality in our study cohort (aOR 0.69, 95% CI 0.38–1.26, *p* = 0.227).

**Table 2:** Multivariate analysis of the predictors of the primary study endpoints (cerebral infarction, in-hospital mortality and unfavorable outcome)

Parameter	aOR (95% CI)	*p*-value
*Cerebral infarcts*		
THRT	0.64 (0.41 – 0.99)	**0.045**
Age (per-year-increase)	1.01 (0.99 – 1.02)	0.209
Sex (female)	1.04 (0.78 – 1.39)	0.805
WFNS grade 4–5	2.71 (2.02 – 3.64)	** < 0.0001**
Fisher grade 3–4	1.09 (0.68 – 1.75)	0.719
Acute hydrocephalus	1.95 (1.38 – 2.75)	** < 0.0001**
Aneurysm rebleeding	3.07 (1.57 – 5.99)	**0.001**
Clipping	1.47 (1.11 – 1.94)	**0.007**
*In-hospital mortality*		
THRT	0.69 (0.38–1.26)	0.227
Age (per-year-increase)	1.04 (1.02 – 1.05)	** < 0.0001**
Sex (female)	0.94 (0.64 – 1.38)	0.764
WFNS grade 4–5	3.84 (2.58 – 5.73)	** < 0.0001**
Fisher grade 3–4	3.15 (0.97 – 10.26)	0.057
Acute hydrocephalus	1.39 (0.83 – 2.34)	0.215
Aneurysm rebleeding	4.27 (2.36 – 7.73)	** < 0.0001**
Clipping	1.03 (0.70 – 1.50)	0.886
*Unfavorable outcome (modified Rankin scale* > *3 6 months after SAH)*		
THRT	0.50 (0.28 – 0.89)	**0.018**
Age (per-year-increase)	1.05 (1.04–1.07)	** < 0.0001**
Sex (female)	0.63 (0.43 – 0.93)	**0.022**
WFNS grade 4–5	7.04 (4.90 – 10.11)	** < 0.0001**
Fisher grade 3–4	2.38 (0.91 – 6.18)	0.073
Acute hydrocephalus	2.11 (1.33 – 3.35)	**0.002**
Aneurysm rebleeding	4.75 (2.26 – 9.98)	** < 0.0001**
Clipping	2.06 (1.47 – 2.89)	** < 0.0001**

Abbreviations: *SAH* subarachnoid hemorrhage, *aOR* adjusted Odds Ratio, *THRT* thyroid hormone replacement therapy due to pre-existing hypothyroidism, *TCD* transcranial Doppler sonography, *WFNS* World Federation of Neurosurgical Societies. Significant findings in bold

Of the secondary study endpoints, the occurrence of ischemia-relevant cerebrovascular events (adjusted unstandardized coefficient -0.14 per-new-event, 95% CI -0.02 – -0.26, *p* = 0.026) and fever in the course of disease (aOR 0.56, 95% CI 0.33–0.96, *p* = 0.036) were significantly and independently related to THRT. A subgroup analysis on fever (with or without systemic infections, see supplementary table S4) and other secondary endpoints of the study did not show significant results in the multivariate analysis (Table 3).

**Table 3:** Multivariate analysis of the predictors of the secondary study endpoints

Parameter	aOR/aUC (95% CI)	*p*-value
*Ischemia-relevant cerebrovascular events (summary of ICP-/vasospasm-related events)*		
THRT	-0.14 (-0.02 – -0.26)	**0.026**
Age (per-year-increase)	-0.01 (-0.01 – -0.01)	** < 0.0001**
Sex (female)	0.06 (-0.03 – 0.14)	0.183
WFNS grade 4–5	0.06 (-0.02 – 0.15)	0.133
Fisher grade 3–4	0.11 (-0.02 – 0.23)	0.085
Acute hydrocephalus	0.28 (0.18 – 0.37)	** < 0.0001**
Aneurysm rebleeding	0.33 (0.15 – 0.51)	** < 0.0001**
Clipping	0.28 (0.20 – 0.36)	** < 0.0001**
*Shunt dependency*		
THRT	0.92 (0.55 – 1.53)	0.756
Age (per-year-increase)	0.99 (0.99 – 1.01)	0.652
Sex (female)	1.11 (0.79 – 1.57)	0.560
WFNS grade 4–5	1.75 (1.26 – 2.44)	** < 0.0001**
Fisher grade 3–4	1.89 (0.93 – 3.81)	0.076
Acute hydrocephalus	8.56 (5.00 – 14.65)	** < 0.0001**
Aneurysm rebleeding	1.50 (0.72 – 3.12)	0.278
Clipping	1.14 (0.82 – 1.57)	0.444
*Fever*		
THRT	0.56 (0.33 – 0.96)	**0.036**
Age (per-year-increase)	1.01 (0.99 – 1.03)	0.078
Sex (female)	0.62 (0.41 – 0.93)	**0.021**
WFNS grade 4–5	1.59 (1.02 – 2.47)	**0.037**
Fisher grade 3–4	1.73 (1.08 – 2.75)	**0.022**
Acute hydrocephalus	4.37 (2.89 – 6.60)	** < 0.0001**
Aneurysm rebleeding	0.42 (0.21 – 0.85)	**0.015**
Clipping	2.90 (1.89 – 4.45)	** < 0.0001**
*Systemic infections*		
THRT	1.10 (0.70 – 1.73)	0.689
Age (per-year-increase)	1.00 (0.99 – 1.01)	0.452
Sex (female)	0.73 (0.54 – 0.99)	**0.041**
WFNS grade 4–5	1.82 (1.34 – 2.46)	** < 0.0001**
Fisher grade 3–4	2.17 (1.22 – 3.86)	**0.009**
Acute hydrocephalus	1.85 (1.28 – 1.47)	**0.001**
Aneurysm rebleeding	0.82 (0.46 – 1.47)	0.502
Clipping	1.76 (1.31 – 2.35)	** < 0.0001**

Abbreviations: *SAH* subarachnoid hemorrhage, *aOR* adjusted Odds Ratio, *aUC* adjusted unstandardized coefficient, *THRT* thyroid hormone replacement therapy due to pre-existing hypothyroidism, *WFNS* World Federation of Neurosurgical Societies. Significant findings in bold

## Discussion

Our data suggest that in patients with a ruptured intracranial aneurysm THRT may be protective. There was a significant inverse association between THRT and the risk of ischemia-relevant cerebrovascular events related to ICP-increase and cerebral vasospasms. Fittingly, SAH patients with THRT showed a lower incidence of cerebral infarcts throughout the disease course. This association was independent in the multivariate analysis. In the long-term clinical follow-up, THRT was additionally linked to a better functional outcome.

Thyroid hormones play pivotal roles in numerous physiological and pathophysiological processes within the central nervous and other organ systems [22]. They mediate blood circulation by exerting positive chronotropic and ionotropic effects on the heart, along with reducing systemic vascular resistance [4]. Additionally, thyroid hormones directly influence glucose and lipid metabolism, protein synthesis, and play crucial roles in immune system function by modulating neutrophil and macrophage activity [4, 14].

Within the central nervous system, thyroid hormones possess genomic (regulation of mitochondrial energetics, modulation of specific gene expression, etc.) and nongenomic (protein trafficking, cell migration, mediation of ion transport, etc.) effects [5]. Experimental and clinical studies indicate that thyroid hormones exhibit neuroprotective actions in ischemic brain injury, such as reducing infarct burden and facilitating postischemic remodeling, leading to improved neurological outcomes [5]. Other potentially neuroprotective effects of thyroid hormones include enhancing glucose transport into brain cells [37], promoting brain connectivity [5], and mitigating glutamate toxicity [28]. Furthermore, thyroid hormones have been shown to increase brain vasculature density [38] and reduce post-ischemic brain edema [27] in preclinical settings.

During acute pathologic conditions, such as sepsis, myocardial infarction, stoke or after complex surgical procedures necessitating prolonged intensive care treatment, a substantial portion of individuals (according to a recent large meta-analysis, about 58% of patients [43]) develop a condition defined as nonthyroidal illness syndrome (NTIS), also known as the euthyroid sick syndrome or the sick euthyroid syndrome. NTIS is characterized by decreased serum thyroid hormone levels without a corresponding increase in thyroid-stimulating hormone secretion. NTIS has a multifactorial nature, including hormone inhibition by cytokines, free fatty acids, bilirubin, and various drugs used in the intensive care unit, downregulation of thyroid hormone receptors, and low concentrations of binding proteins [14, 47]. Several studies have highlighted a significant inverse correlation between serum levels of interleukins and thyroid hormones [4, 11, 29, 35], underscoring the role of inflammation in NTIS pathogenesis [44].

NTIS has been significantly associated with high complication rates and poor prognoses across various diagnostic groups. A recent meta-analysis confirmed NTIS as an independent predictor of mortality in critically ill patients [43]. Reduced concentrations of thyroid hormones may be implicated in altered cardiac inotropism and chronotropism, altered vasoactive properties, diminished cognitive status with lethargy, inability to control bacterial infections, respiratory muscle weakness, reduced synthesis of pulmonary surfactant, and decrease of lung compliance resulting in the impairment of lung function [43].

As previously mentioned, thyroid hormones are crucial for normal brain function and reparative processes following acute brain injury. Reduced cerebral perfusion has been observed in individuals with low free T4 levels, indicating an inverse association between thyroid hormones and global cerebral perfusion [12]. Congruent results were demonstrated in a clinical trial performed in healthy euthyroid volunteers, where a mild induced thyrotoxicosis led to a better cerebellar perfusion and function [15]. Meta-analyses of studies on ischemic stroke have consistently shown strong associations between NTIS and/or low thyroid hormone levels and greater baseline stroke severity, poorer functional outcome, and higher overall mortality [5, 20, 23].

The occurrence of NTIS and its association with poor functional outcome was also reported in the context of SAH [6, 25, 47]. However, the messages derivable from these studies were strongly limited by the sample sizes of the analyzed cohorts (20–35 patients) that necessitated further clinical evaluation. An experimental SAH study supported the findings from clinical trials and showed that thyroid hormone treatment promotes mitophagy, decreases microglial activation, alleviates neuroinflammation, and reduces neuronal apoptosis following SAH [8].

While the literature on the effects of thyroid dysfunction and THRT on SAH is sparse, the efforts to explain the impact of thyroid hormones on the cascades in the brain after aneurysm rupture remain speculative. The functional relevance of thyroid hormones and the risk of NTIS after acute conditions like SAH offer a plausible explanation for our findings.

Despite being largely overlooked in the clinical management of SAH patients, NTIS poses a significant risk following SAH. Therefore, providing exogenous thyroid hormone supplementation may help address the thyroid hormone deficiency observed in SAH patients with NTIS. This increased availability of thyroid hormones may confer systemic and neuroprotective benefits, including reducing the burden of oxidative stress and cellular damage due to mitochondrial dysfunction, downregulation of neuro-inflammation and apoptosis, and more pronounced repair mechanisms. Hence, THRT might substantially contribute to lower rate of complications and poor outcome observed in our study.

Notably, SAH patients receiving THRT demonstrated reduced incidences of ICP- and vasospasm-related cerebrovascular events, as well as a trend toward less severe brain edema on admission CT scans. These results align with previous reports on the impact of thyroid hormones on brain edema risk [27], and the density and contractility of intracranial vasculature [38]. Thus, thyroid hormones might help to reduce the extent of brain edema after SAH, and lower the risk of subsequent ICP increase.

Additionally, SAH patients receiving THRT experienced fewer febrile episodes during their hospital stay. As this association was not related to the occurrence of systemic infections in our analysis, THRT-dependent fever risk after SAH might be rather of central genesis. Suppression of neuroinflammation by thyroid hormones, as reported in experimental studies [5, 8], may contribute to this effect. In turn, less prominent neuro-inflammation and fever under THRT can positively impact the risk of cerebral vasospasm and infarction after SAH. The substantial body of clinical and experimental literature underlining the importance of inflammation [2, 9, 24] and fever [21, 26, 33] for development of vasospasm and infarct support this assumption.

In the intensive care setting, THRT during SAH may enhance hemodynamic stability by maintaining adequate cardiac output and reducing systemic vascular resistance. Therefore, it can be argued that THRT can promote perfusion in the acutely damaged brain tissue after SAH. As a result, improved cerebral perfusion would lower the risk of DCI and poor outcome after SAH. Indeed, individuals with THRT had less cerebral infarctions and unfavorable outcome at 6 months after SAH independent of major outcome confounders.

Our study underscores the clinical relevance of the associations between THRT and the occurrence of complications and poor outcomes in SAH. It highlights the essential role of thyroid metabolism in the pathophysiological processes of SAH and positions thyroid hormones as promising therapeutic agents for reducing brain damage and improving patient outcomes. However, further studies are warranted to confirm whether THRT benefits are specific to SAH patients with NTIS or extend to those with intact thyroid metabolism. Additionally, prospective studies are needed to elucidate the biological mechanisms underlying the observed associations between THRT and SAH outcomes. Future research could consist of a prospective study that firstly evaluates the associations between the baseline thyroid hormones levels as well as the fluctuations in these levels with the course and outcome of SAH in a large representative cohort. A detailed previous history concerning thyroid diseases and medication should be obtained via the patient, their relatives/care takers and general practitioners. Depending on the thyroid metabolism at the onset and during SAH and at the absence of other contraindications, a low dosage THRT could be applied in an interventional arm of a prospective randomized controlled clinical trial, designed preferably in a double-blind fashion to minimize bias.

The major drawback of this study is its retrospective design, limiting the possibility of detailed elucidation of thyroid function prior to the SAH event. Moreover, serum measurements of thyroid hormones at admission are lacking. On the other side, an acute bleeding event might strongly impact the hormone release limiting the interpretability of a routine thyroid hormone screening at admission in an eventual prospective study. Furthermore, we could not verify if the protective effect is due to the structural and functional changes in the brain (and, probably, in the whole organism) related to the pre-existing hypothyroid metabolism or rather to THRT, which SAH patients were taking during the acute phase of disease.

## Conclusions

In this first study evaluating the value of THRT in the course of SAH, individuals with exogenous thyroid hormones administration showed lower burden of cerebrovascular adverse events and lower risk of cerebral infarction and poor outcome. Further experimental and clinical studies are required to confirm our results and highlight the background of the eventual impact of THRT on the course and outcome of SAH.

## Supplementary Information

Below is the link to the electronic supplementary material.

Supplementary file1 (DOCX 22.3 KB)

## Data Availability

Available upon reasonable request via email to the first author (said.maryam@gmail.com).
