# Supplementary material for: Impact of thyroid hormone replacement therapy on the course and functional outcome of aneurysmal subarachnoid hemorrhage
Source: Acta Neurochir (Wien). 2024 Jun 3;166(1):245. doi: 10.1007/s00701-024-06118-7 (PMC11147837; doi:10.1007/s00701-024-06118-7)
Supplement: Supplementary file 1 — Supplementary file1 (DOCX 22.3 KB) [file 701_2024_6118_MOESM1_ESM.docx]

**ONLINE SUPPLEMENTS**

**Supplementary Table S1: Variables with missing data**

| **Parameter** | **Number (%)** | **Reason** |
| --- | --- | --- |
| Previous medical history | 2 (0.2%) | Missing records |
| Radiographic data (SEBES & Fisher grading) | 106 (10.7%) | Initial imaging from other hospitals not stored in our institutional PACS |
| DIND | 161 (16.2%) | Non-assessable due to continuously poor neurological condition |
| TCD vasospasms | 142 (14.3%) | Non-assessable due to insufficient acoustic window |
| Shunt dependency | 150 (15.1%) | Death or transferal to another clinic prior to completing of EVD weaning |
| Fever | 85 (8.5%) | Missing or incompletely collected data from the electronic health records |
| Systemic infections | 100 (10%) |  |
| Cerebral infarcts | 7 (7%) | No follow-up CT imaging |
| Unfavorable outcome^*^ | 69 (6.9%) | Lost to follow-up |

Abbreviations: SAH= aneurysmal subarachnoid hemorrhage, SEBES= Subarachnoid hemorrhage Early Brain Edema Score, PCAS = picture archiving and communication system, DIND = delayed ischemic neurological deficit, TCD= transcranial Doppler sonography, EVD= external ventricular drain, CT= computed tomography. * =, † = modified Rankin scale >3 6 months after ictus.

**Supplementary Table S2: Comparison of baseline characteristics of SAH individuals with and without aneurysm treatment**

| **Parameter** | **Number (%**^*^**) or Mean (±SD)** | | **OR (95% CI)** | **p-value** |
| --- | --- | --- | --- | --- |
|  | **No treatment (n=51)** | **Treatment (n=944)** |  |  |
| Age (years) | 66 (±14) | 54 (±14) | - | **<0.0001** |
| Female | 33 (64.7%) | 634 (67.2%) | 1.12 (0.62 – 2.01) | 0.760 |
| WFNS grade 4-5 | 39 (76.5%) | 374 (39.6%) | 0.20 (0.10 – 0.39) | **<0.0001** |
| SEBES grade 3-4 | 24 (53.3%) | 403 (47.7%) | 0.80 (0.44 – 1.46) | 0.541 |
| Fisher grade 3-4 | 46 (92%) | 707 (85.9%) | 0.53 (0.19 – 1.50) | 0.292 |
| Rebleeding | 16 (31.4%) | 42 (4.4%) | 0.10 (0.05 – 0.20) | **<0.0001** |
| Acute hydrocephalus | 35 (68.6%) | 659 (69.8%) | 1.06 (0.58 – 1.94) | 0.876 |

Abbreviations: OR= odds ratio, CI = confidence interval, SAH= aneurysmal subarachnoid hemorrhage, WFNS= World Federation of Neurosurgical Societies, SEBES= Subarachnoid hemorrhage Early Brain Edema Score; * = percentages were calculated according to the number of cases with known values. Significant findings in **bold**.

**Supplementary Table S3: Comparison of baseline characteristics of SAH individuals with and without THRT due to pre-existing hypothyroidism: unadjusted univariate analyses**

| **Parameter** | **Number (%**^*^**) or Mean (±SD)** | | **OR (95% CI)** | **p-value** |
| --- | --- | --- | --- | --- |
|  | **No THRT (n=886)** | **THRT (n=109)** |  |  |
| *Demographics* | | | | |
| Age (years) | 54.4 (±14.0) | 57.5 (±14.0) | - | **0.013** |
| Female | 569 (64.2%) | 98 (89.9%) | 4.96 (2.62 – 9.40) | **<0.0001** |
| *Initial SAH and aneurysm characteristics* | | | | |
| WFNS grade 4-5 | 368 (41.5%) | 45 (41.3%) | 0.99 (0.66 – 1.48) | 1.000 |
| SEBES grade 3-4 | 389 (49.0%) | 38 (39.6%) | 0.68 (0.44 – 1.05) | 0.085 |
| Fisher grade 3-4 | 667 (86.1%) | 86 (87.8%) | 1.16 (0.61 – 2.20) | 0.756 |
| Clipping | 328 (39.0%) | 37 (35.9%) | 0.88 (0.57 – 1.34) | 0.593 |
| Rebleeding | 50 (5.6%) | 8 (7.3%) | 1.32 (0.61 – 2.87) | 0.513 |
| Acute hydrocephalus | 611 (69.0%) | 83 (76.1%) | 1.44 (0.90 – 2.28) | 0.150 |
| *Complications after SAH* | | | | |
| ICP increase >20 mmHg | 397 (45.2%) | 43 (40.6%) | 0.83 (0.55 – 1.25) | 0.409 |
| DC | 232 (26.2%) | 25 (22.9%) | 0.84 (0.52 – 1.34) | 0.562 |
| DIND | 232 (31.2%) | 24 (26.1%) | 0.78 (0.48 – 1.27) | 0.340 |
| TCD vasospasms | 398 (52.2%) | 44 (48.4%) | 0.86 (0.55 – 1.32) | 0.507 |
| Shunt dependency | 248 (32.9%) | 31 (33.7%) | 1.04 (0.66 – 1.64) | 0.907 |
| Fever  Fever with systemic infections^†^  Fever without systemic infections^†^ | 649 (79.8%)  301 (39.3%)  315 (41.2%) | 71 (71.7%)  39 (41.9%)  30 (32.3%) | 0.64 (0.40 – 1.03)  1.11 (0.72 – 1.72)  0.68 (0.43 – 1.08) | 0.068  0.654  0.117 |
| Systemic infections | 332 (41.7%) | 43 (43.4%) | 1.08 (0.71 – 1.64) | 0.747 |
| *Outcome after SAH* | | | | |
| Cerebral infarcts | 433 (49.2%) | 44 (40.7%) | 0.71 (0.47 – 1.07) | 0.103 |
| In-hospital mortality | 163 (18.4%) | 17 (15.6%) | 0.82 (0.48 – 1.41) | 0.513 |
| Unfavorable outcome^§^ | 321 (38.9%) | 28 (28.0%) | 0.61 (0.39 – 0.97) | **0.038** |

Abbreviations: OR= odds ratio, CI = confidence interval, SAH= aneurysmal subarachnoid hemorrhage, THRT= thyroid hormone replacement therapy due to pre-existing hypothyroidism, WFNS= World Federation of Neurosurgical Societies, SEBES= Subarachnoid hemorrhage Early Brain Edema Score, DC= Decompressive craniectomy, ICP= intracranial pressure, DIND = delayed ischemic neurological deficit, TCD= transcranial Doppler sonography. * = percentages were calculated according to the number of cases with known values; † = calculated upon the cases with available documentation on fever and systemic infections; § = modified Rankin scale >3 6 months after ictus. Significant findings in **bold**.

**Supplementary Table S4. Multivariate analysis of the predictors of the secondary study endpoints not reported in the main paper**

| **Parameter** | **aOR (95 % CI)** | **p-value** |
| --- | --- | --- |
| ***Fever with systemic infections*** | | |
| THRT | 1.10 (0.66 – 1.85) | 0.712 |
| Age (per-year-increase) | 1.00 (0.99 – 1.02) | 0.379 |
| Sex (female) | 0.68 (0.45 – 1.02) | 0.063 |
| WFNS grade 4-5 | 1.82 (1.27 – 2.60) | **0.002** |
| Fisher grade 3-4 | 2.52 (1.05 – 6.07) | **0.041** |
| Acute hydrocephalus | 1.34 (0.85 – 2.11) | 0.201 |
| Aneurysm rebleeding | 0.92 (0.50 – 1.71) | 0.797 |
| Clipping | 1.60 (1.12 – 2.29) | **0.011** |
| ***Fever without systemic infections*** | | |
| THRT | 0.66 (0.34 – 1.30) | 0.217 |
| Age (per-year-increase) | 1.00 (0.99 - 1.01) | 0.892 |
| Sex (female) | 1.02 (0.73 – 1.44) | 0.901 |
| WFNS grade 4-5 | 0.74 (0.50 – 1.09) | 0.115 |
| Fisher grade 3-4 | 0.88 (0.46 – 1.71) | 0.691 |
| Acute hydrocephalus | 1.65 (1.09 – 2.50) | **0.018** |
| Aneurysm rebleeding | 0.77 (0.43 – 1.39) | 0.388 |
| Clipping | 1.06 (0.80 – 1.42) | 0.682 |

Abbreviations: SAH= subarachnoid hemorrhage, aOR= adjusted Odds Ratio, THRT= thyroid hormone replacement therapy due to pre-existing hypothyroidism, WFNS= World Federation of Neurosurgical Societies. Significant findings in **bold**.
